# Transcriptome profiling of high and low somatic embryogenesis rate of oil palm (*Elaeis guineensis* Jacq. var. Tenera)

**DOI:** 10.3389/fpls.2023.1142868

**Published:** 2023-05-12

**Authors:** Asri Sahara, Roberdi Roberdi, Ni Made Armini Wiendi, Tony Liwang

**Affiliations:** ^1^ Biotechnology Department, Plant Production and Biotechnology Division, PT SMART Tbk, Bogor, Indonesia; ^2^ Agronomy and Horticulture Department, Agriculture Faculty, Bogor Agricultural University, Bogor, Indonesia

**Keywords:** differentially expressed genes, globular embryoid, high embryogenic, oil palm, somatic embryogenesis, RNA-seq

## Abstract

Oil palm micropropagation through tissue culture is a technique to provide elite oil palms to meet the desired traits. This technique is commonly carried out through somatic embryogenesis. However, the oil palm’s somatic embryogenesis rate is quite low. Several approaches have been made to overcome this problem, including transcriptome profiling through RNA-seq to identify key genes involved in oil palm somatic embryogenesis. RNA sequencing was applied in high- and low-embryogenic ortets of Tenera varieties based on the somatic embryoid rate at the callus, globular, scutellar, and coleoptilar embryoid stages. Cellular analysis of embryoid inductions and proliferations showed that high-embryogenic ortets resulted in higher embryoid proliferation and germinations than low-embryogenic ortets. Transcriptome profiling showed that there are a total of 1,911 differentially expressed genes (DEGs) between high- and low-embryogenic ortets. ABA signaling-related genes such as *LEA*, *DDX28*, and *vicilin-like protein* are upregulated in high-embryogenic ortets. Furthermore, DEGs associated with other hormone signaling, such as *HD-ZIP* associated with brassinosteroids and *NPF* associated with auxin, are upregulated in high-embryogenic ortets. This result suggests a physiological difference between high- and low-embryogenic ortets that is connected to their capacity for somatic embryogenesis. These DEGs will be used as potential biomarkers for high-embryogenic ortets and will be validated in further studies.

## 1 Introduction

As the most productive oil-bearing plant in the world (FAO, 2021), improvement of oil palm planting materials is necessary to fulfill the high demand for this vegetable oil. One of the techniques that can be applied to improve oil palm planting material is tissue culture. *In vitro* clonal seedling techniques can increase yield between 20% and 30% in comparison to conventional seedlings. This increase is due to the use of high-yielding ortets as an explant source (Corley & Tinker, 2003) and true-to-type plants that are genetically identical to their ortets (Shearman et al., 2013). Oil palm is a monocotyledon plant that only has one shoot apical meristem (Weckx et al., 2019). Oil palm *in vitro* clonal seedling techniques are carried out through somatic embryogenesis. However, the oil palm somatic embryogenesis rate is only 1%–5% (Roowi et al., 2010). Somatic embryogenesis is affected by several factors, including genotype, mother plant, media type, and explant source (Constantin et al., 2015). The plant genotype is a main factor in the oil palm embryogenic competence (Roowi et al., 2010). Somatic embryogenesis involves various physiological, cellular, and molecular activities, including dedifferentiation and reprogramming of gene expression patterns (Karami & Saidi, 2010).

Transcriptomics is an RNA transcript used for the study of gene expression differences and gene pathways in activating or suppressing several genes during somatic embryogenesis (Rajesh et al., 2016). Several genes related to oil palm embryogenesis have been identified using molecular analyses. For example, using expressed sequence tags, two genes, namely, *lipid-transfer protein (LTP)* and *glutathione S-transferase (GST)*, were differentially expressed between embryogenic and non-embryogenic callus (Low et al., 2008). Moreover, using microarray and quantitative real-time PCR (qPCR), some oil palm somatic embryogenesis genes, i.e., *IAA-amino acid hydrolase ILR1-like 1 (ILR1)* and *late embryogenesis abundant (LEA2)*, were also detected as biomarkers to differentiate the embryoid stage from the callus stage (Syariyanto et al., 2018). Recently, transcriptome profiling of leaves of high- and low-embryogenic ortets revealed that flowering-related genes such as *flowering locus T-interacting protein (FTIP)*, *frigida-like (FRL)*, and *nuclear transcription factor y subunit A-7 (NF-YA)* were upregulated in high-embryogenic ortets (Ooi et al., 2021). However, the study of RNA transcripts in the somatic embryoid development of high- and low-embryogenic oil palm is not yet available. Moreover, somatic embryogenesis encompasses three developmental stages consisting of embryonic induction, embryonic, and developmental stages (Elhiti et al., 2013). Therefore, it is important to analyze the molecular mechanisms of somatic embryoids not only in the induction stages but also in the embryonic and developmental stages.

RNA sequencing enables high throughput of RNA transcript analysis through cDNA sequencing and can provide quantitative information on gene expression and differentially expressed genes (DEGs) (Kukurba & Montgomery, 2015). Thus, this technique allows the identification of the key genes involved in oil palm somatic embryogenesis through analysis of DEGs between high- and low-embryogenic ortets. In this study, RNA sequencing was used to obtain a transcriptome profile of the high- and low-embryogenic ortets at the callus and somatic embryoid stages. This study examined the DEGs between the high- and low-embryogenic ortets at the callus and several embryoid stages. Functional gene identification related to embryogenesis is expected to facilitate the decision-making process in improving the efficiency of large-scale oil palm *in vitro* propagation.

## 2 Materials and methods

### 2.1 Plant materials and culture condition

**Figure 1:**
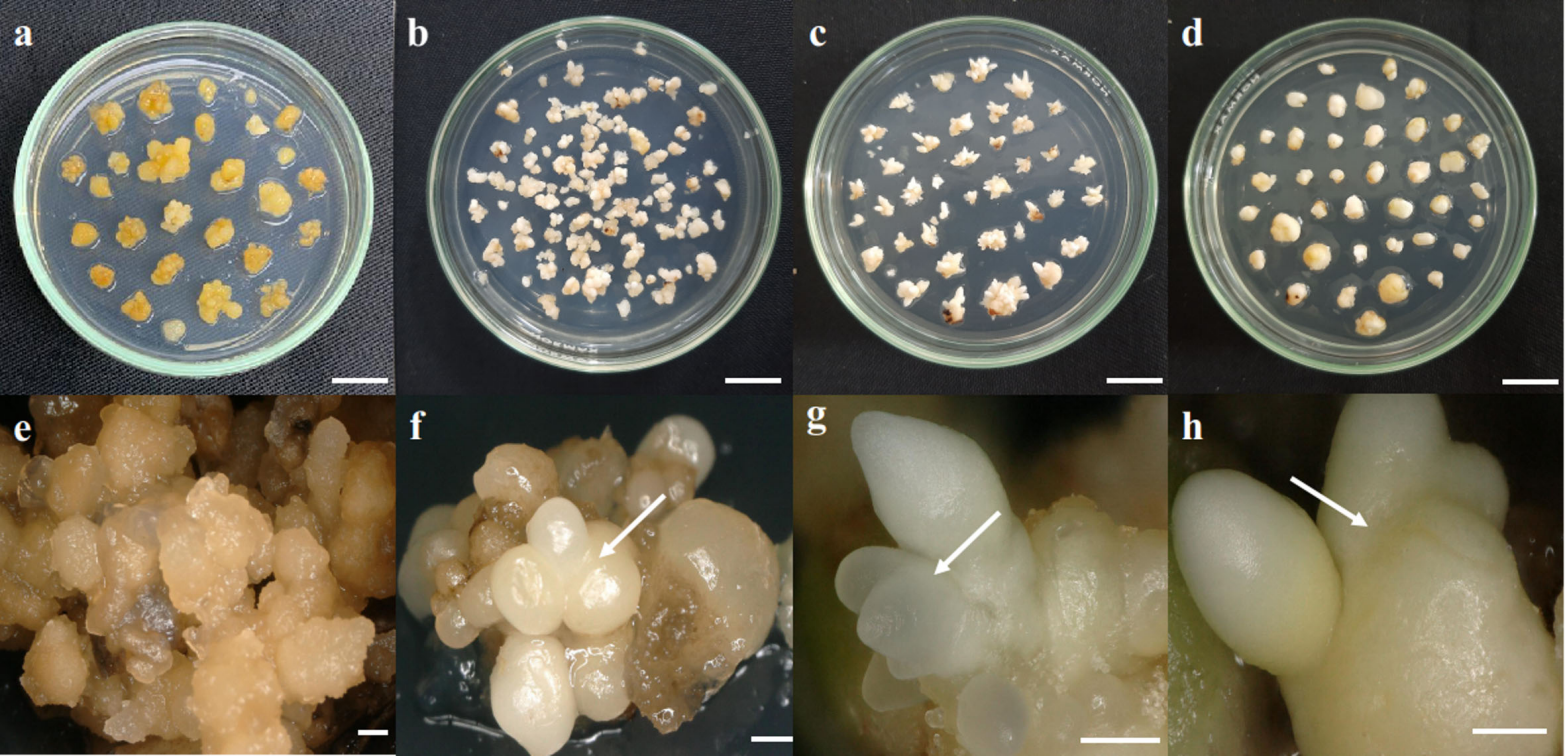
Somatic embryoid development stages. **(A, E)** Callus, **(B, F)** globular embryoid, **(C, G)** scutellar embryoid, and **(D, H)** coleoptilar embryoid. Scale bars: **(A–D)**, 1 cm; **(E)**, 1 mm; **(F–H)**, 0.5 mm.

The callus and three somatic embryoid development stages consisting of globular, scutellar, and coleoptilar (**Figure 1**) from four Tenera (DxP) mother palms from Deli Dura origin with high oil productivity (8–9 tons·ha^−1^·year^−1^) were used. Mother palms were coded as ortets 10818-r, 20818-r, 10119-t, and 10319-t. The ortet somatic embryoid rates performances were used as criteria for the ortet selection. The average somatic embryoid rate was 1% and used as an internal benchmark (PT. SMART Tbk, 2020). There were ortets that showed distinct somatic embryoid performance and were selected for this study. Ortets 10119-t and 10319-t were the highest embryogenic ortets, whereas ortets 10818-r and 20818-r were the lowest embryogenic ortets. Callus and somatic embryoid were obtained from immature leaves that were cultured in Murashige and Skoog medium (Murashige & Skoog, 1962) with the addition of 5% sucrose and 0.65% agar. Callus induction was conducted by subculturing immature leaf explants every 3 months during the 12 months of the incubation period, while callus was subcultured every 2 months during the 12 months of the incubation period to induce the somatic embryoid. In total, 3,500 immature leaf explants with a size of 1 × 1 cm from each ortet were cultured in callus induction media. Cellular analyses were conducted to measure the callus formation rate, somatic embryo formation rate, somatic embryo proliferation rate, and germination rate. The callus formation rate was calculated from the number of clump calli per explant, while the somatic embryo rate was calculated from the number of somatic embryoids per clump callus in each ortet. Embryoid proliferations were performed by subculturing the first clump of each somatic embryoid line every 2 months in proliferation and germination media. Embryoids were cultured in a 100-ml Erlenmeyer flask containing 15 clumps of 3–5-mm proliferated embryoid. The proliferation and germination rates of each embryoid line per subculture period were calculated based on the frequency of proliferated and germinated cultures against the number of initial cultures in the first subculture. The germinated embryoid was the embryoid that had formed at least one bud and leaf.

### 2.2 RNA extraction

The identification of callus and somatic embryoid development was conducted using a Keyence VHX-6000 stereo microscope (Keyence, Osaka, Japan). The somatic embryoid of each stage was separated from each other, consisting of callus, globular, scutellar, and coleoptilar phases. Total RNA was extracted from the callus and three somatic embryoid stages using the RNeasy^®^ Plant Mini Kit (Qiagen, Hilden, Germany) and following the manufacturer’s instructions. The total RNA of the callus was extracted from three biological replicates for each ortet. Each replication was collected by bulking five callus lines from the sixth subculture, resulting in a total of 12 samples. The total RNA of each somatic embryoid development and each ortet was extracted from three biological replicates. The first and second replications were obtained from different embryoid lines, while the third replication was obtained by bulking two embryoid lines of the first and second replications. The embryoids of each stage and ortet were collected from several flasks from the fourth to seventh subcultures derived from the same embryoid line. Thus, the total sample of embryoid stages was 36. In total, there were 48 observation units for RNA-seq. The purity of the RNA was examined using a NanoDrop™2000c Spectrophotometer (Thermo Scientific, Massachusetts, United States), and the *RNA integrity number* (RIN) was examined using a QC 2100 Bioanalyzer Instrument (Agilent Company, Santa Clara, CA, United States).

### 2.3 Bioinformatics analysis of cDNA sequence data

RNA sequencing was conducted by Novogene Co., Ltd., Beijing, China. Total RNA was used for cDNA synthesis through reverse transcriptase polymerase chain reaction (RT-PCR) for cDNA library construction. The libraries were sequenced by Illumina HiSeq 4000 (Illumina, San Diego, CA, United States). The reads were mapped to the *Elaeis guineensis* transcriptome reference (GCF_000442705.1_EG5) in the National Center for Biotechnology Information (NCBI) (www.ncbi.nlm.nih.gov). Transcripts per million (TPM) and the number of expressed genes were counted using Kallisto software (Bray et al., 2016). Identification of DEGs with |log_2_fold change| > 1 and p-value < 0.05 was performed through pairwise comparisons between high- and low-embryogenic samples at each stage using DESeq2 (Love et al., 2014). DEG enrichment and gene ontology were analyzed by PlantRegMap software (Tian et al., 2020).

## 3 Results

### 3.1 Cellular analysis of callus and somatic embryoid

**Figure 2:**
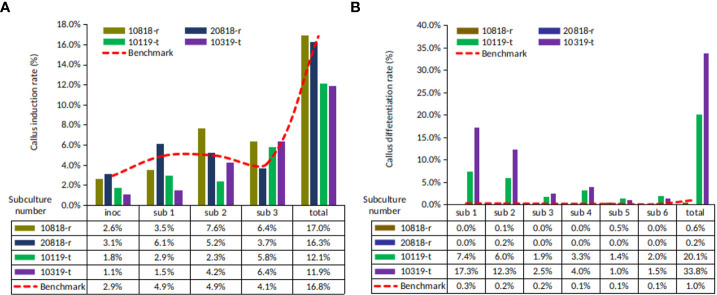
Callus and somatic embryoid rate from oil palm leaf explant of four ortets. **(A)** Callus induction rate. **(B)** Somatic embryoid rate. Inoc, inoculation; sub-1, first subculture; sub-2, second subculture; sub-3, third subculture, etc.

The callus induction (CI) rates of four ortets ranged from 11.9% to 17.0% (**Figure 2A**). Ortets 10119-t and 10319-t showed a lower CI rate than the internal benchmark of 16.8% (PT. SMART Tbk, 2020). Ortets 10818-r and 20818-r showed CI rates of 17.0% and 16.3%, respectively. However, their somatic embryoid rates were only 0.6% and 0.2%, respectively (**Figure 2B**). Ortets 10119-t and 10319-t showed high somatic embryogenesis rates at 20.1% and 33.8%, respectively, which are higher than the internal benchmark of 1.0% (PT. SMART Tbk, 2020). Based on these performances, ortets 10119-t and 10319-t were categorized as high-embryogenic palms, while ortets 10818-r and 20818-r were categorized as low-embryogenic palms. The callus induction rate of the four ortets did not show a significant difference. Significant differences from the four ortets are shown in the callus differentiation stage to form somatic embryos. These results indicated that there were differences in the callus differentiation process between high- and low-embryogenic ortets that affected embryogenesis. Thus, a molecular analysis of the callus stage was needed to reveal differences in callus differentiation between the two ortet categories.

**Figure 3:**
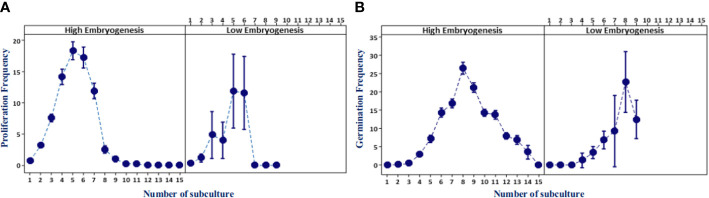
Embryoid proliferation and germination frequency of high- and low-embryogenic ortets. **(A)** Embryoid proliferation. **(B)** Embryoid germination. Two ortets represent the high embryogenesis category, ortets 10119-t and 10319-t. Two ortets represent the low embryogenesis category, ortets 10818-r and 20818-r.

Somatic embryoid proliferation growth during subculture formed a sigmoid curve (**Figure 3A**). The embryoid proliferation of all ortets was increased gradually until the fifth subculture and decreased continually until the 15th subculture. High-embryogenic ortets (ortets 10119-t and 10319-t) showed a higher embryoid proliferation than low-embryogenic ortets (ortets 10818-r and 20818-r). The embryoid of high-embryogenic ortets proliferated until the 15th subculture, whereas the low-embryogenic ortets only proliferated until the 10th subculture. At the fifth subculture, high-embryogenic ortets showed the highest embryoid proliferation, increasing 18-fold from the starting point. Meanwhile, the highest embryoid proliferation of the low-embryogenic ortets was only 12-fold. The somatic embryoids reached the maturation stage and then germinated into shoots at the fourth subculture. The highest germination rates from high- and low-embryogenic ortets were observed in the eighth subculture at 25-fold and 22-fold, respectively (**Figure 3B**). The differences in embryoid proliferation and germination between high- and low-embryogenic ortets indicated differences in embryoid development. These results indicated the requirement for a molecular analysis of embryoid development to reveal the differences between the two ortet categories.

### 3.2 Callus and embryoid transcriptome profiles according to embryogenic category

**Figure 4:**
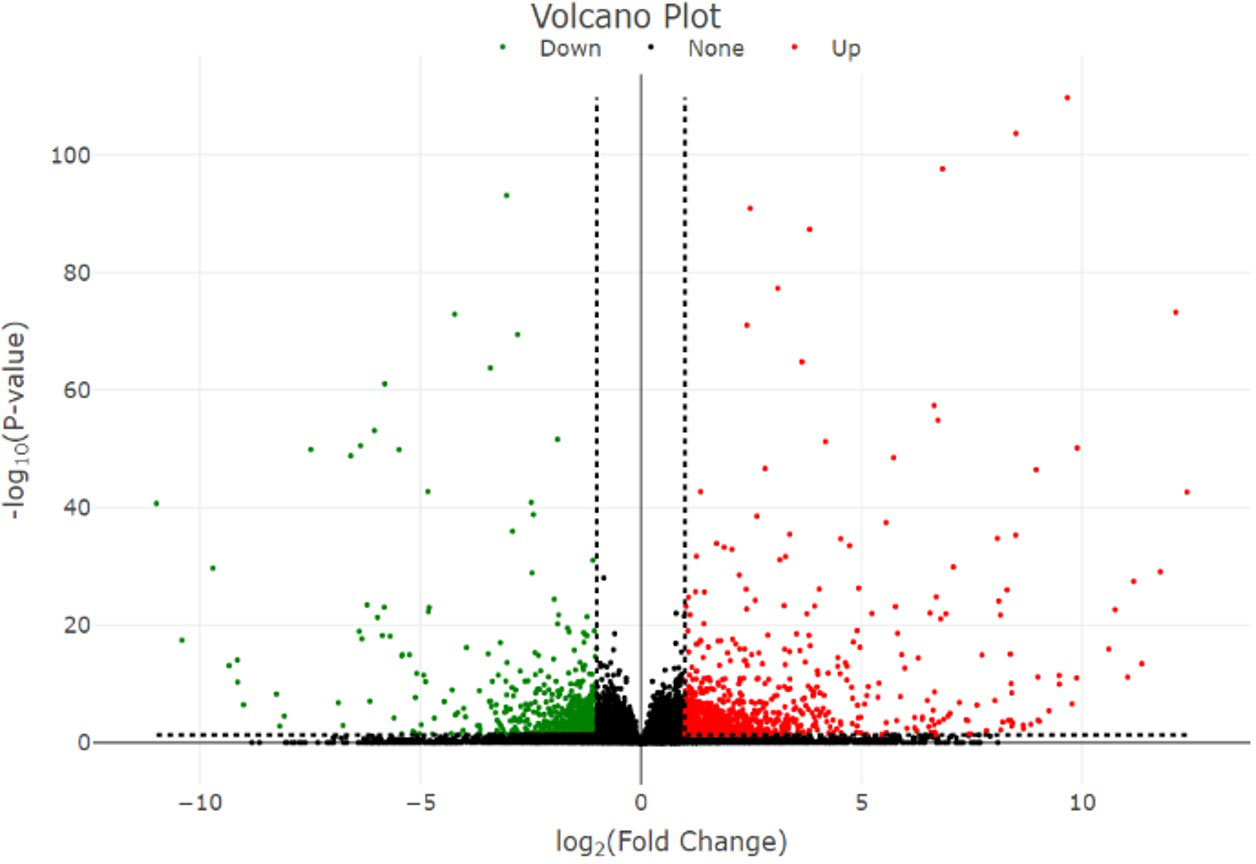
Volcano plot of the DEGs. Volcano plot represents the distribution of significant genes. The y-axis represents the significant level of gene expression between samples measured by the p-value, while the x-axis represents the fold change (log_2_ fold change) of DEGs between high- and low-embryogenic samples. DEGs, differentially expressed genes.

In total, 1,911 DEGs were identified between high- and low-embryogenic ortets at the callus and embryoid stages. The DEG distribution was presented in a volcano plot (**Figure 4**). Gene outer line |log_2_FC| > 1 and p-value < 0.05 were considered to be differentially expressed. As far as the distance of the DEG distribution from the |log_2_FC| line, the log2 fold change value will be higher. The p-value showed the significance level of the DEGs transcript to the total transcript of all samples, which described the transcript abundance of DEGs compared to the total transcript. Meanwhile, the log_2_FC value showed the level of DEG fold change between samples.

**Figure 5:**
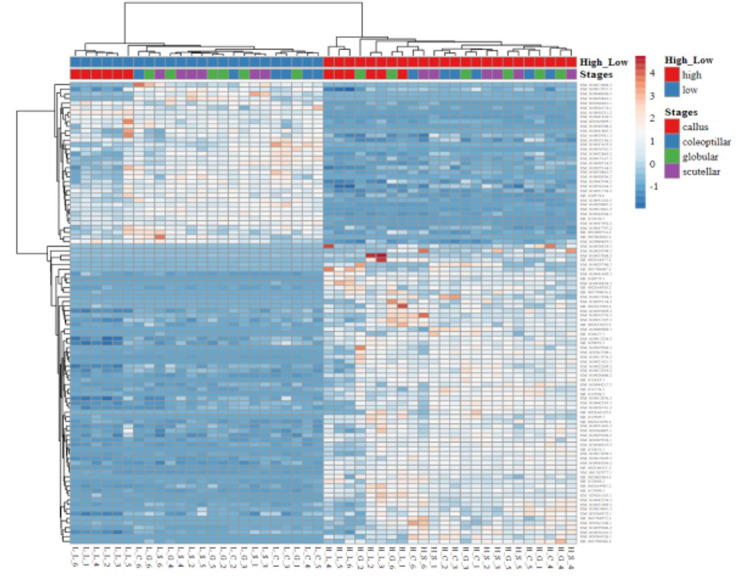
Heatmap of the DEGs. Heatmap of gene expression profiles of high- and low-embryogenic samples based on the transcript per million (TPM) of top 100 DEGs based on p-value. DEGs, differentially expressed genes.

**Figure 6:**
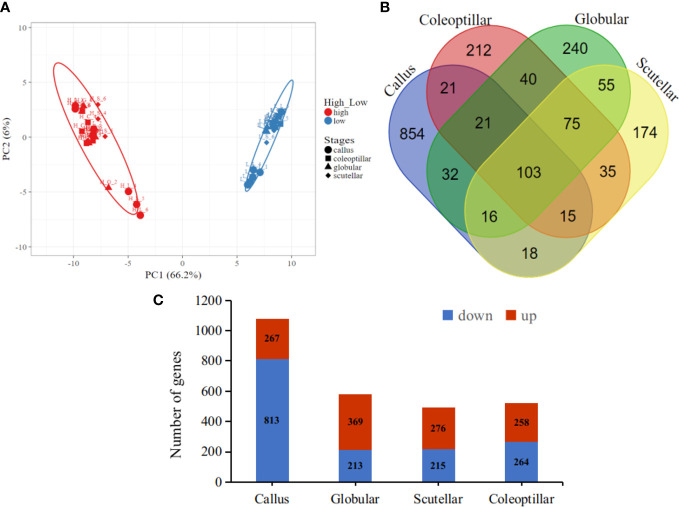
Transcriptome profiles of the DEGs. **(A)** PCA of the callus and somatic embryoid based on the transcript per million (TPM) of DEG, high embryogenesis (red) and low embryogenesis (blue). **(B)** Venn DEG diagrams of four somatic embryogenesis stages. **(C)** The number of upregulated and downregulated DEGs at each embryogenesis stage. DEGs, differentially expressed genes; PCA, principal component analysis.

The gene expression in high- and low-embryogenic ortets is shown in the heatmap (**Figure 5**). The heatmap displays two major clusters on the x-axis. The first cluster contained low-embryogenic ortets, while the second contained high-embryogenic ortets. One group of DEGs exhibited higher expression in the high-embryogenic ortets but lower expression in the low-embryogenic ortets, while the other group of DEGs showed higher expression in the low-embryogenic ortets but lower expression in the high-embryogenic ortets. This result demonstrated the differences in transcriptome profiles of high- and low-embryogenic ortets at the callus and embryoid stages. Principal component analysis (PCA) also grouped the sample into two major groups, i.e., the high embryogenesis group (red) and the low embryogenesis group (blue), based on the number of transcripts per million DEGs (**Figure 6A**). Callus was grouped separately from globular, scutellar, and coleoptilar embryoid. This result indicated that transcriptome profiles of embryoid development were more similar to each other and different from the callus transcriptome profile.

In total, 103 DEGs were expressed in all somatic embryogenesis stages. Furthermore, 854, 240, 174, and 212 DEGs were specifically expressed in the callus, globular, scutellar, and coleoptilar stages, respectively (**Figure 6B**). The callus stage had the highest number of DEGs when compared to the other stages. Most DEGs at the callus stage were downregulated in high-embryogenic ortets, while most DEGs at embryoid development stages were upregulated in high-embryogenic ortets (**Figure 6C**). This result indicated that the transcriptome profile of the somatic embryoid induction stage was different from that of the somatic embryoid development stage. This result showed that more genes were involved at the embryoid induction stages than at the embryoid developmental stages.

### 3.3 Functional classification based on gene ontology

The DEGs of high- and low-embryogenic ortets were grouped into 425 gene ontology (GO), consisting of 49 cellular components (11.5%), 131 molecular functions (30.8%), and 245 biological processes (57.6%) (**Table 1**). The “single organism process” GO term (GO:0044699) showed the highest number of genes in the biological processes category, followed by “single-organism metabolic process” (GO:0044710) and “metabolic process” (GO:0008152). The “response to stimulus” GO term (GO:0050896) was a sub-category that also showed a high number of genes. This pattern suggests that the appearance of “response to stimulus” GO is in accordance with the environmental factors during the tissue culture process that involved various physical and chemical stimuli such as light, temperature, humidity, and media hormone concentration.

**Table 1:** Top 30 GO based on the number of DEGs in the three GO categories.

Aspect	GO.ID	Term	Callus		Globular		Scutellar		Coleoptilar		Total
			Down	Up	Down	Up	Down	Up	Down	Up	
Biological process	GO:0044699	Single-organism process	230								230
	GO:0044710	Single-organism metabolic process	124	37							161
	GO:0008152	Metabolic process		85	61						146
	GO:0050896	Response to stimulus	113								113
	GO:0055114	Oxidation–reduction process	63			30					93
	GO:0006950	Response to stress	78								78
	GO:1902578	Single-organism localization	58								58
	GO:0044765	Single-organism transport	57								57
	GO:0071704	Organic substance metabolic process			51						51
	GO:0044238	Primary metabolic process			49						49
	GO:0006807	Nitrogen compound metabolic process						42			42
	GO:0034641	Cellular nitrogen compound metabolic process						39			39
	GO:0043170	Macromolecule metabolic process			38						38
	GO:0044260	Cellular macromolecule metabolic process			36						36
Molecular function	GO:0003824	Catalytic activity	267			107					374
	GO:0016491	Oxidoreductase activity	68			31					99
	GO:0005515	Protein binding			42				53		95
	GO:0005488	Binding			85						85
	GO:0043168	Anion binding		35					36		71
	GO:0000166	Nucleotide binding			31				36		67
	GO:1901265	Nucleoside phosphate binding			31				36		67
	GO:0097367	Carbohydrate derivative binding			29				32		61
	GO:0005215	Transporter activity	44								44
	GO:0016787	Hydrolase activity				38					38
	GO:0003676	Nucleic acid binding						36			36
Cellular component	GO:0016020	Membrane	104								104
	GO:0005622	Intracellular		60							60
	GO:0071944	Cell periphery	50								50
	GO:0005886	Plasma membrane	42								42
	GO:0016021	Integral component of membrane	37								37
	GO:0031224	Intrinsic component of membrane	37								37

GO, gene ontology; DEGs, differentially expressed genes.

### 3.4 Differentially expressed gene of callus and somatic embryoids of high- and low-embryogenic ortets

**Figure 7:**
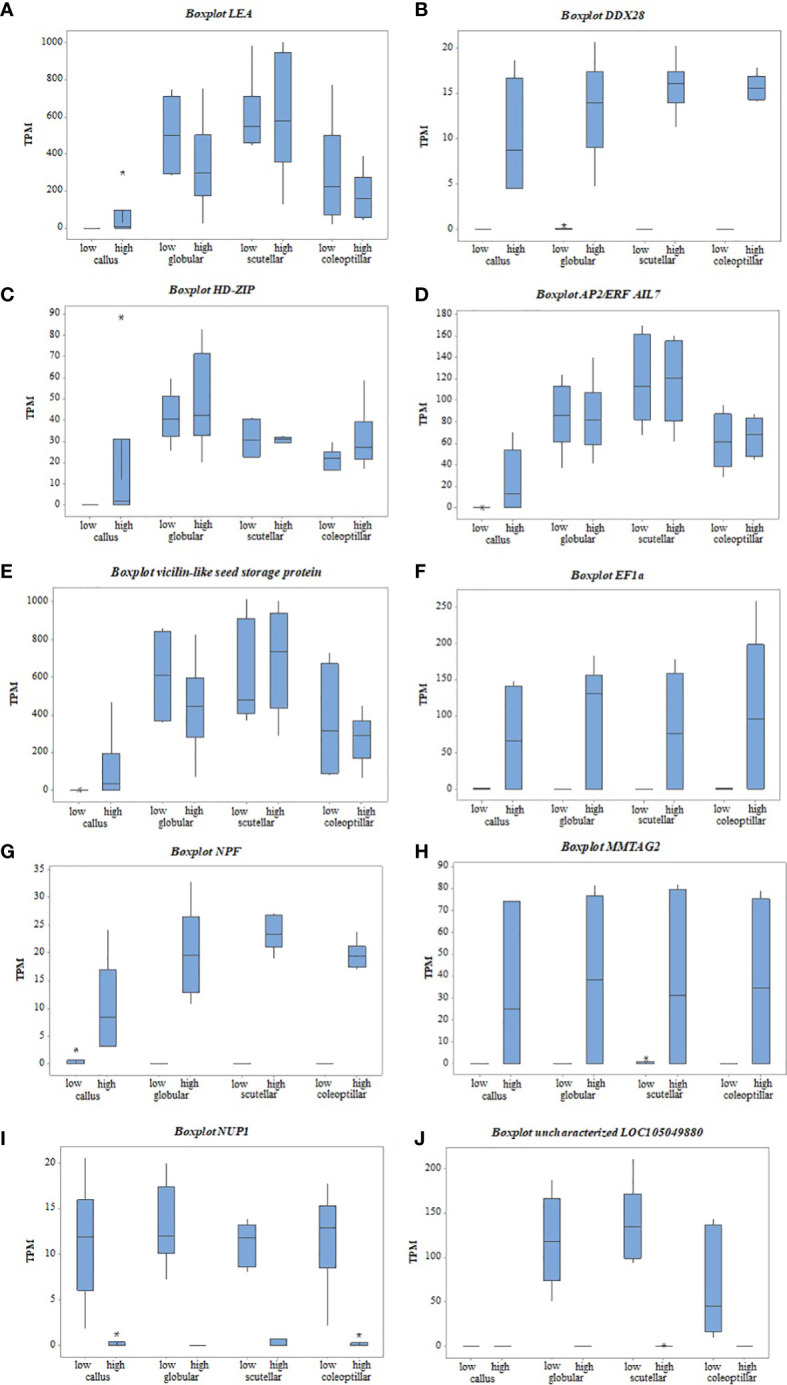
The gene expression of top DEGs between high- and low-embryogenic ortets at callus and embryoid stages based on TPM values. **(A)** LEA gene, **(B)** DDX28 gene, **(C)** HD-ZIP gene, **(D)** AP2/ERF AIL7 gene, **(E)** Vicilin-like seed gene, **(F)** EF1a gene, **(G)** NPF gene, **(H)** MMTAG2 gene, **(I)** NUP1 gene, **(J)** uncharacterized LOC105049880 gene. DEGs, differentially expressed genes; TPM, transcript per million.

The gene candidates involved in oil palm somatic embryogenesis were determined by the highest value of |log_2_FC| of DEGs and their physiological function (**Table 2**). The top DEGs based on |log_2_FC| were *elongation factor 1-alpha (EF1a)*, *vicilin-like seed storage protein At2g28490*, *nuclear pore complex protein (NUP1)*, *uncharacterized LOC105049880*, *protein NRT1/PTR FAMILY 8.3 (NPF)*, *multiple myeloma tumor-associated protein 2 homolog (MMTAG2)*, *AP2-like ethylene-responsive transcription factor AIL7 (AP2/ERF AIL7)*, *late embryogenesis abundant (LEA)*, *DEAD-box ATP-dependent RNA helicase 28 (DDX28)*, and *homeobox-leucine zipper protein ROC2* (*HD-ZIP*). The expression of these genes based on the TPM value showed a significant difference between high- and low-embryogenic ortets (**Figure 7**). The expression level of the top genes was varied, but most of them were upregulated in high-embryogenic ortets, i.e., *EF1a*, *vicilin-like protein*, *NPF*, *MMTAG2*, *AP2/ERF AIL7*, *LEA*, *DDX28*, and *HD-ZIP*. In contrast, the two genes were downregulated, i.e., *NUP1* and *uncharacterized LOC105049880*.

**Table 2:** Top 25 DEGS based on the |log_2_FC| value for each somatic embryogenesis development.

LOC ID	Annotation	Maximum value of |log_2_FC|	log_2_FC*			
			Callus	Globular	Scutellar	Coleoptilar
LOC105058399	Elongation factor 1-alpha	13,263		8,613	13,263	8,057
LOC105044822	Vicilin-like seed storage protein At2g28490	12,450	12,450			
LOC105042431	Nuclear pore complex protein NUP1	12,364	−6,247	−12,364	−5,598	−5,94
LOC105049880	Uncharacterized LOC105049880	12,027		−9,799	−9,558	−12,027
LOC105043992	Protein NRT1/PTR FAMILY 8.3	11,852		11,852	11,76	11,478
LOC105061430	Multiple myeloma tumor-associated protein 2 homolog	11,729	10,805	11,729		11,347
LOC105056440	AP2-like ethylene-responsive transcription factor AIL7	11,625	11,625			
LOC105042293	Late embryogenesis abundant protein D-34	11,568	11,568			
LOC105044056	DEAD-box ATP-dependent RNA helicase 28	11,542	10,612	7,624	11,542	11,523
LOC105035130	Homeobox-leucine zipper protein ROC2	11,453	11,453			
LOC105042491	Aquaporin TIP3-1	11,429	11,429			9,797
LOC105049086	Obg-like ATPase 1	11,309		11,039		11,309
LOC105050755	Ubiquitin receptor RAD23b	11,262	11,262			8,312
LOC105048583	Uncharacterized LOC105048583	11,261		11,261		
LOC105058627	RAN GTPase-activating protein 1	10,834	−10,834	−5,409	−5,065	−5,507
LOC105058627	RAN GTPase-activating protein 1	10,765	10,173	10,765	6,42	10,476
LOC105060596	Kinesin-like protein KIN-14L	10,744	10,744			2,339
LOC105034731	B2 protein	10,716	10,203	10,706	10,716	6,363
LOC105042294	Late embryogenesis abundant protein D-34	10,576	10,576			
LOC105035093	Protein FAR1-RELATED SEQUENCE 6	10,485		−10,485	−4,955	
LOC105043740	Ubiquitin carboxyl-terminal hydrolase 3	10,465	8,946	10,465	9,892	9,797
LOC105055797	Transcription factor UNE12	10,347	−6,29	−10,347	−5,427	−7,447
LOC105059952	Cell division cycle protein 123 homolog	10,305	9,718	10,264	9,961	10,305
LOC105040320	Uncharacterized LOC105040320	10,251				10,251
LOC105046508	Protein TIME FOR COFFEE	10,238		10,026		10,238

*The positive value (+) represents the upregulated expression in high-embryogenic sample, and the negative value (−) represents the downregulated expression in high-embryogenic sample.

Transcriptome analysis of callus revealed that several top DEGs were upregulated in the high-embryogenic ortets and absent in the low-embryogenic ortets, i.e., *LEA*, *DDX28*, and *HD-ZIP* (**Figures 7A-C**). The expression of *LEA* increased gradually from callus to globular, scutellar, and coleoptilar (**Figure 7A**). It could be suggested that *LEA* was more involved in somatic embryoid development than in embryoid induction. However, the expression of this gene was lacking in the callus of the low-embryogenic ortets. Hence, it might indicate that this gene is necessary for embryogenic induction. The absence of *DDX28* and *HD-ZIP* expression (**Figures 7B, C**) in the callus of low-embryogenic ortets may influence the embryogenic potential, but this requires more detailed investigation. In this study, *HD-ZIP* was expressed in all stages of somatic embryoid development in both embryogenic categories but was absent in the callus of low-embryogenic ortets.

The top DEGs that were upregulated in the high-embryogenic ortets but downregulated in the low-embryogenic ortets were *AP2/ERF*, *vicilin-like protein*, *EF1*, *NPF*, and *MMTAG2*. The expression of *AP2/ERF* and *vicilin-like protein* continually increased from callus to globular, scutellar, and coleoptilar stages in either high- or low-embryogenic ortets, but the significant difference only occurred in the callus stage (**Figures 7D, E**). This pattern suggested that *AP2/ERF* and *vicilin-like protein* were involved in all stages of the somatic embryoid, but the low expression of this gene in the callus stage might result in low-embryogenic induction.

The identification of the DEGs in the embryoid development stages, i.e., globular, scutellar, and coleoptilar, was necessary to analyze the low proliferation and germination of embryoids in low-embryogenic ortets. *EF1a*, *NPF*, and *MMTAG2* were identified as DEGs at callus and embryoid development. These genes were upregulated in the high-embryogenic ortets and downregulated in the low-embryogenic ortets at callus and embryoid stages (**Figures 7F-H**). The downregulation of these genes in the low-embryogenic ortets might influence not only embryoid induction but also embryoid development, i.e., embryoid proliferation and germination. Meanwhile, there were two downregulated DEGs in the high-embryogenic ortets, i.e., *NUP1* and *uncharacterized LOC105049880* (**Figures 7I, J**).

## 4 Discussion

Calli were formed from leaf explants through cell dedifferentiation. The callus induction rate of four ortets ranged from 11.9% to 17.0%. The significant difference between the four ortets was shown in callus differentiation to form somatic embryoids. Two ortets showed high embryogenesis performance, i.e., 10119-t and 10319-t; the other two ortets showed low embryogenesis performance, i.e., 10818-r and 20818-r. Callus differentiation involves the transition from callus cells to stem-like cells that will initiate embryoids (Rose et al., 2022). Auxin gradients at the transition stage from callus to embryonic stem-like cells promoted embryonic cell induction (Chen et al., 2022). This process stimulated transcription factors for early embryoid development that induced the activation of somatic embryo-related genes.

The expression of several DEGs was upregulated in the high-embryogenic ortets and absent in the low-embryogenic ortets i.e., *LEA*, *DDX28*, and *HD-ZIP* (**Figures 7A-C**). *LEA* was also identified as DEGs between embryogenic and non-embryogenic calli by microarray (Budinarta et al., 2012). The expression of this gene was validated with q-PCR and showed that the *LEA* expression was higher at the embryoid development stages than at the callus stage (Syariyanto et al., 2018), and we obtained a similar result in this study. Another study revealed that *LEA* plays an important role in zygotic as well as somatic embryogenesis through ABA treatment (Ikeda et al., 2006). A recent study on oil palm leaf transcriptome profiling reported that *DDX28* was also differentially expressed between high- and low-embryogenic ortets (Ooi et al., 2021). Based on *in silico* analysis in rice, the member of *DDX28* interacted with the pathway of ABA phytohormone signaling (Macovei et al., 2012). In addition to auxin, ABA was the predominant phytohormone that affected embryogenesis potential. In wheat and barley, somatic embryogenesis potential is determined by the level of indoleacetic and abscisic acids (Seldimirova et al., 2019). In the oil palm, *DDX28* is also involved in inflorescence sex determination (Ho et al., 2016). In another research, *HD-ZIP* was reported to be highly expressed during early oil palm somatic embryogenesis (Ooi et al., 2016). *HD-ZIP* is the transcription factor that has a role in various plant growth functions, plant adaptation to several environmental stressors, and plant growth regulator pathways (Hong et al., 2021). In *Arabidopsis*, *HD-ZIP* is a transcription factor that controls the regulation of *brassinosteroid-related-homeobox 3 (BHB3)* . Brassinosteroid is a phytohormone that plays an important role as a plant growth regulator (Hasegawa et al., 2022). Based on the results, our study showed that most of the DEGs were associated with phytohormone regulation, especially with ABA.

Some DEGs were expressed in the callus of high-embryogenic ortets but downregulated in the callus of low-embryogenic ortets, i.e., *AP2/ERF* and *vicilin-like protein* (**Figures 7D, E**). *AP2/ERF* and *vicilin-like protein* were involved in all stages of somatic embryoids. However, the low expression of this gene in the callus of low-embryogenic ortets might result in low-embryogenic induction. A transcription factor from *apetala2 (AP2)* family, such as *BABY BOOM (BBM)*, plays an important role in cell proliferation and induction of somatic embryogenesis in *Arabidopsis thaliana* and *Brassica napus* (Boutilier et al., 2002). Another study reported that *AP2/ERF* is involved in various primary metabolic processes as well as secondary metabolic processes, growth and development of the plant, and response to environmental stress (Licausi et al., 2013). Based on *Basic Local Alignment Search Toll* (BLAST) data, *AP2-ERF AIL7* protein in NCBI showed a functional domain of AP2-type that was similar to BBM family in *A. thaliana* and *B. napus* with homology of 75.21% and 80.95%, respectively. The involvement of the *AP2* family in embryogenic callus and somatic embryo development was also reported in oil palm (Morcillo et al., 2007). Moreover, in *Arabidopsis*, overexpression of the *AINTEGUMENTA-LIKE (AIL)* transcription factors promotes embryogenesis and organogenesis (Magnani et al., 2017). A similar pattern with *AP2/ERF* expression was exhibited by *vicilin-like protein* gene. *Vicilin-like protein* encodes a storage protein located in the chloroplast as well as cytoplasm (Van de Vondel et al., 2020) that is found in cereal seeds and nuts (Burrieza et al., 2019). *Vicilin-like proteins* play roles in seed growth and response to external stress (Ma et al., 2022). *Vicilin-like protein* is also necessary for LEA protein coding for seed germination that was provided by ABA (Hu & Ma, 2006).

Some DEGs were significant not only in the callus stage but also in embryoid development, i.e., *EF1a*, *NPF*, and *MMTAG2*. The downregulation of these genes in the low-embryogenic ortets might influence not only embryoid induction but also embryoid development, i.e., embryoid proliferation and germination. Another study reported that *EF1a* was expressed during somatic embryogenesis and germination of the *Liriodendron* hybrid (Li et al., 2021). The involvement of *EF1a* in somatic embryo maturation was also reported in Norway spruce (*Picea abies*) (Wang-Lougheed et al., 2004). *EF1a* is a member of the GTP binding protein (guanine nucleotide-binding protein) that plays a role in protein synthesis, actin filaments, and microtubules during the cell cycle (Li et al., 2021). The main function of this protein was to transport tRNA to the ribosome. Furthermore, *NPF* plays a role in the transport of various substrates, including nitrates and several hormones. *NPF* acts as a nitrate sensor that affects auxin transport. The auxin transport by *NPF* is inhibited by certain nitrate concentrations (Corratgé-Faillie & Lacombe, 2017). In *Arabidopsis*, *NPF* plays a role in the nitrogen content of the somatic embryo and is highly expressed at the maturation stage (Leran et al., 2015). The nitrogen content influences the protein content in the somatic embryoid of *Glycine max* (L.) (Truong et al., 2013). Then, the protein content determined the quality of the mature somatic embryoid of *Medicago sativa* (L.) (Lai & Mckersie, 1994). Another study reported that nitrogen supply affected the germination of the somatic embryoid of *P. abies* (Carlsson et al., 2018). The function of *MMTAG2* in oil palm in addition to being related to somatic embryogenesis is not well characterized. *MMTAG2* is reported to be involved in the mechanism of resistance to gummy stem blight (GSB) disease and is used as a marker-assisted selection in melon plant breeding (Han et al., 2022). *MMTAG2* causes DNA imbalance and encourages the activation of DNA repair pathways caused by the production of reactive oxygen species (ROS) (Saitoh & Oda, 2021). ROS are involved in embryogenesis during the dedifferentiation of somatic cells into callus cells (Rose et al., 2022).

Two DEGs were downregulated in high-embryogenic ortets and upregulated in low-embryogenic ortets, i.e., *NUP1* and *uncharacterized LOC105049880*. *NUP* is a nuclear membrane protein that regulates ABA in order to respond the abiotic stress (Zhu et al., 2017). *NUP1* is necessary for miRNA transport from the nucleus to the cytoplasm. The decrease in *NUP1* expression resulted in the inhibition of the miRNA transport (Zhang et al., 2020). *NUP1* interacts with some locus in the chromosome that could inhibit, activate, and regulate the expression of several genes (Sumner & Brickner, 2022). The *nuclear pore complex (NPC)* mutant was reported to inhibit the somatic embryo development in *Arabidopsis*. It was because the signaling pathway of some phytohormones was sensitive to the interference of *NPC* (Wu et al., 2022). The function of *uncharacterized LOC105049880* gene in addition to being related to somatic embryogenesis is not well characterized.

The interaction of appropriate genotypes and the expression of specific genes from each embryogenesis stage promote embryoid induction. The biological stages of embryogenic induction consisted of dedifferentiation, embryogenic stem cells, and early embryo development (Rose et al., 2022). Each step involved a specific gene that supports somatic embryogenesis. The transition of callus cells to stem-like cells required an auxin gradient and involved genes related to auxin signaling and transport (Chen et al., 2022). In this study, *NPF*, a gene related to auxin transport, was identified as DEGs between high- and low-embryogenic ortets. The differential expression of auxin transport genes might contribute to the determination of auxin levels between high- and low-embryogenic ortets. In *Medicago truncatula*, the auxin gradient was described as high in early callus development and proliferation but degraded at the transition of callus to embryogenic stem cells (Chen et al., 2022). Auxin induced the expression of genes that modified the genetic program of somatic cells and regulated the transition of somatic cells to somatic embryo development (Loyola-Vargas & Ochoa-Alejo, 2016). The use of auxins such as 2,4-D was reported to increase the expression of a transcription factor for somatic embryo induction (Shivani et al., 2017).

The degradation of auxin levels in the transition of callus to embryogenic stem cells and the increase of ABA in this stage promoted high somatic embryogenesis (Chen et al., 2022). Several DEGs were related to ABA signaling, which were *LEA*, *DDX28*, *vicilin-like protein*, and *NUP1*. ABA has an important role in the induction of somatic embryoids. Several plants required exogenous ABA in the medium to enhance the somatic embryoid (Ikeda et al., 2006; Méndez-Hernández et al., 2019). ABA displayed additive effects on promoting cell fate transition from callus cells to embryogenic stem cells (Chen et al., 2022). The application of ABA in oil palm tissue culture is reported to increase the maturation of somatic embryos (Teixeira et al., 1994; Bertossi et al., 2001; Jayanthi et al., 2011). However, its application in somatic embryo induction has not been reported. In this study, several DEGs were associated with ABA signaling in the callus stage. This result indicated that ABA is involved in not only embryoid development but also embryoid induction. The addition of ABA to embryoid induction media and analysis of endogenous content in the callus stage of high- and low-embryogenic ortets could be used to investigate the role of ABA in the induction of oil palm somatic embryos.

The transition of callus to embryogenic required transcription factors for further embryoid development. In this study, transcription factors *AP2/ERF* and *HD-ZIP* were identified as DEGs between high and low in the embryoid induction stages. Transcription factors of embryogenesis were very important in the somatic embryoid induction (Chu et al., 2017) and affected the expression of the somatic embryoid-related gene (Méndez-Hernández et al., 2019). The interaction of appropriate hormones and transcription was required for the embryonic stem cells to develop into embryos (Rose et al., 2022).

## 5 Conclusion

Cellular analysis at explant and callus stages showed that the embryogenesis rates of high-embryogenic ortets 10119-t and 10319-t were 20.1% and 33.8%, respectively, but the low-embryogenic ortets 10818-r and 20818-r were only 0.6% and 0.2%, respectively. High-embryogenic ortets have higher somatic embryoid proliferation and germination rates at 18-fold and 25-fold, respectively, but the low-embryogenic ortets were only 12-fold and 22-fold, respectively. Transcriptome analysis through RNA sequencing showed that the high-embryogenic ortets had a different transcriptome profile from the low-embryogenic ortets at callus and somatic embryoid stages based on the heatmap and PCA. A total of 103 DEGs were expressed in all embryogenesis stages; 854 DEGs were specifically expressed in the callus stage, 240 DEGs in the globular stage, 174 DEGs in the scutellar stage, and 212 DEGs in the coleoptilar stage. Embryogenesis-related gene candidates were selected based on the top |log_2_FC| value and their physiological function. Gene candidates were commonly associated with the regulation of hormones such as ABA, auxins, and brassinosteroids. The role of each gene indicates a physiological state associated with the potential for somatic embryogenesis. The top DEGs need to be validated in further studies.

## Data availability statement

The original contributions presented in the study are included in the article/supplementary material, further inquiries can be directed to the corresponding author.

## Author contributions

AS, RR, NW and TL designed the research. AS performed the research and wrote the manuscript. All authors contributed to the article and approved the submitted version.
